# Potential therapies for acute‐on‐chronic liver failure

**DOI:** 10.1111/liv.15545

**Published:** 2023-03-08

**Authors:** Maura A. Morrison, Florent Artru, Francesca M. Trovato, Evangelos Triantafyllou, Mark J. McPhail

**Affiliations:** ^1^ Institute of Liver Studies King's College Hospital London UK; ^2^ Department of Inflammation Biology, School of Immunology and Microbial Sciences King's College London London UK; ^3^ Section of Hepatology and Gastroenterology, Department of Metabolism, Digestion and Reproduction Imperial College London London UK

## Abstract

Acute‐on‐chronic liver failure (ACLF) is a syndrome that develops in approximately 30% of patients hospitalised with cirrhosis and is characterised by an acute decompensation of liver function associated with extra‐hepatic organ failures and a high short‐term mortality. At present, no specific therapies are available for ACLF, and current management is limited to treatment of the precipitating event and organ support. Given the high prevalence and high mortality of this severe liver disease, there is an urgent need for targeted treatments. There is increasing evidence of the important role played by systemic inflammation and immune dysfunction in the pathophysiology of ACLF and a better understanding of these immune processes is resulting in new therapeutic targets. The aim of this review is to present an overview of ongoing studies of potentially promising therapies and how they could be utilised in the management of ACLF.

**Key points**

- ACLF is a high mortality complication of chronic liver disease, characterised by an acute decompensation of liver function with one or more extra‐hepatic organ failures.
- Several novel therapies targeting immune dysfunction in ACLF are being explored in varying stages of development from studies in vitro to clinical trials.
- The ideal therapy for ACLF should reduce systemic inflammation without resulting in immune exhaustion or hypo‐reactivity to bacterial challenge.
- ACLF patients are a heterogenous population, with variations in aetiology of cirrhosis, precipitating event and organ failure profile, which should be considered when developing novel therapies or updated disease definitions.

## 1 INTRODUCTION

Acute‐on‐chronic liver failure (ACLF) is a syndrome that develops in patients with chronic liver disease, characterised by an acute decompensation of liver function, one or more extra‐hepatic organ failures and a high short‐term mortality rate.^1^, ^2^ Prevalence is high with ACLF developing in about 30% of patients hospitalised for an acute decompensation of cirrhosis.^1^ According to the European Association for the Study of the Liver (EASL) definition, ACLF is classified into three grades based on the number of organ failures with higher grades associated with an increased risk of 28‐day mortality, varying from 23% to 77% in grades 1 to 3 respectively.^1^ The development of ACLF is often precipitated by events such as bacterial infection, gastrointestinal haemorrhage, alcoholic hepatitis, relapsed chronic viral hepatitis and surgery.^1^, ^2^ ACLF is considered a dynamic syndrome as it can deteriorate, improve or resolve within the space of a few days. Consequently, the mortality risk is more accurately evaluated after assessment of the final grade of ACLF, calculated between the 3rd and the 7th day of management.^3^

Current treatment of ACLF is largely based on managing the precipitating event, managing associated complications and providing organ support.^2^, ^4^, ^5^ However, these approaches are frequently ineffective, especially in more severe courses of ACLF. Liver transplantation is the only definitive treatment for ACLF and there is a clear survival benefit in highly selected patient groups.^6^, ^7^ Moreover, these patients show acceptable outcomes at 1 year with an observed survival above 80% even in the most severely ill patients.^7^, ^8^, ^9^ Given every new indication of liver transplantation is at the expense of another in the context of organ shortage, it is necessary to develop new approaches to optimise the transplant‐free survival of patients with ACLF.

In recent years, several studies have highlighted the key role played by systemic inflammation and immune dysfunction in the development of ACLF, which provides the rationale for developing immune‐based therapeutic strategies. This approach appears particularly attractive as the modulation of immune system function has recently been associated with improved outcomes in cancer and inflammatory diseases. Immunomodulatory therapies, such as immune checkpoint inhibitors and chimeric antigen receptor (CAR) T cell therapy, have revolutionised the treatment of certain cancers.^10^, ^11^, ^12^, ^13^ Sphingosine 1‐phosphate receptor (S1PR) modulators, which modulate lymphocyte function, reduce relapse rate in multiple sclerosis, and show promise in the treatment of inflammatory bowel diseases in improving remission rates and endoscopic responses.^14^

The aim of this review is to provide a large overview of novel therapeutic targets in preclinical development, as well as treatments already in clinical trials for patients with ACLF. The large majority of these therapies are not currently used in clinical practice and are not yet recommended for use in clinical practice. The stage of development of each therapy mentioned throughout the review is summarised in Table 1. These therapies target a range of different pathways, which can be classified into four different aspects: liver damage and regeneration, systemic inflammation, the gut microbiome and extra‐corporeal and cellular liver support therapies.

**TABLE 1:** Summary of emerging therapies for treatment of acute‐on‐chronic liver failure.

Target	Therapy	Mechanism	Stage	Study Group	Outcome	References
**Liver Damage and Regeneration**						
LPS‐TLR4	TLR4 antagonist (TAK‐242)	Reduces hepatocyte injury	Phase 2 clinical trial	ACLF precipitated by alcoholic hepatitis	Trial closed before recruitment started	NCT04620148
	Recombinant ALP	Deactivates endotoxin	Animal model	LPS‐induced rodent model of ACLF	Pre‐treatment reduces the severity of liver injury, hepatic cytokines and brain oedema	Ref. [15]
	Albumin	Reduces endotoxin activity, reverts PGE2 ‐induced immune dysfunction, inhibits TLR signalling	Clinical	Decompensated cirrhosis	No improvement in survival	Ref. [16]
IL‐1	IL‐1 receptor inhibitor (anakinra)	Inhibits IL‐1‐induced hepatic inflammation	Phase 2 clinical trial	Alcoholic hepatitis	No improvement in survival in the first trial, lower 90‐day survival with anakinra in the second trial	NCT01809132, NCT04072822
	IL‐1*β* inhibitor (canakin‐umab)	Inhibits IL‐1‐induced hepatic inflammation	Phase 2 clinical trial	Alcoholic hepatitis	No improvement in survival or MELD score	NCT03775109
RIPK1	RIPK1 inhibitors (NEC‐1 and RIPA56)	Inhibits RIPK1‐dependent necroptosis	Animal model	ACLF	Pre‐treatment reduces total liver cell death, improves liver and kidney functions in ACLF rodent model	Ref. [17]
IL‐33‐ST2‐CXCR2	CXCR1/2 inhibitors	Reduces hepatocyte death, reduces neutrophil ROS	In vitro	HBV and alcohol‐related ACLF	Not yet studied in animal model/clinical trial	Ref. [18]
	IL‐33	Improves neutrophil migration	In vitro	Alcoholic hepatitis	Not yet studied in animal model/clinical trial	Ref. [19]
TRAIL	TRAIL inhibitor	Reduces apoptosis of hepatocytes	In vitro	HBV ACLF	Not yet studied in animal model/clinical trial	Ref. [20]
NKG2D	NKG2D inhibitor	Reduces inflammatory cytokine production	In vitro	HBV ACLF	Not yet studied in animal model/clinical trial	Ref. [21]
KCTD9	KCTD9 inhibitor	Reduces NK cell cytotoxicity	Animal model	HBV ACLF	Improves survival and liver function, reduces histological liver damage in animal model	Ref. [22, 23]
IL‐22	IL‐22Fc (F‐562)	Promotes hepatocyte proliferation	Phase 2 clinical trial	Alcoholic hepatitis	F‐652 is safe and is associated with improvement in Lille and MELD scores	Refs. [24, 25]
CD34+ stem cells	G‐CSF	Mobilises bone marrow CD34+ haematopoietic stem cells, promotes liver regeneration	Phase 2 clinical trial	All causes of cirrhosis resulting in ACLF, all precipitating events of ACLF	No improvement in survival	Ref. [26]
**Systemic Inflammation**						
TLR7/8	TLR 7/8 inhibitors (CL097, R848)	Restores MPO release, improves bacterial killing	In vitro	Decompensated alcoholic cirrhosis	Not yet studied in animal model/clinical trial	Ref. [27]
MerTK	MerTK inhibitor (UNC569)	Improves LPS‐induced cytokine production, up‐regulates HLA‐DR	In vitro	All aetiologies of ACLF	Not yet studied in animal model/clinical trial	Ref. [28]
AXL	AXL inhibitor (BGB324/ bemcentinib)	Improves LPS‐induced cytokine production	In vitro	All aetiologies of cirrhosis	Not yet studied in animal model/clinical trial	Ref. [29]
Autotaxin‐LPA	LPA	Reduces pro‐regulatory monocyte phenotypes, increases TNFα production	In vitro	All aetiologies of ACLF	Not yet studied in animal model/clinical trial	Ref. [30]
Glutamine synthetase	Glutamine synthetase inhibitor (MSO)	Improves monocyte phagocytic capacity, increases pro‐inflammatory cytokines and reduces anti‐inflammatory cytokines	In vitro	Alcohol‐related ACLF	Not yet studied in animal model/clinical trial	Ref. [31]
TLR3	TLR3 agonist (poly:IC)	Decreases M‐MDSCs, improves phagocytic capacity	In vitro	All aetiologies of ACLF	Not yet studied in animal model/clinical trial	Ref. [32]
PD‐1, PD‐L1, TIM3	PD‐L1 inhibitors	Restores monocyte innate immune responses	Animal model	Model of cirrhosis with added infection	Reduces bacteraemia, prevents impairment in liver injury tests	Ref. [33]
	PD‐1/TIM3 inhibitors	Improves neutrophil phagocytosis and oxidative burst capacities	In vitro	Alcoholic hepatitis	Not yet studied in animal model/clinical trial	Ref. [34]
**Microbiome**						
Bacterial transloca‐tion	Rifaximin/simvastatin	Decrease bacterial translocation and therefore circulating PAMPs	Phase 3 clinical trial	All causes of decompensated cirrhosis	Results awaited	NCT03780673
Bacterial transloca‐tion	NSBBs	Inhibit sympathetic activity, reduce gut bacterial translocation	Observational clinical studies	All causes of cirrhosis resulting in ACLF	Improved survival in patients receiving NSBBs	Refs. [35, 36]
Dysbiosis	Engineered bacteria	Selection of genes for beneficial effects. e.g. IL‐22 producing *Lactobacillus reuteri*	Animal model	Model of alcoholic liver disease	Reduced alcohol‐induced liver injury and inflammation	Ref. [37]
Dysbiosis	FMT	Restores diversity of microbiome	Clinical trial	Alcoholic hepatitis	Improved survival in pilot study, results of RCT awaited	NCT03091010
Specific bacteria	Phage therapy	Lysis of bacteria producing specific enzyme e.g. cytolysin‐producing *E. faecalis*	Animal model	Model of alcoholic hepatitis	Reduced alcohol‐induced liver injury and inflammation	Ref. [38]
**Extra‐Corporeal Liver Support**						
Circul‐ating PAMPs and DAMPs	MARS®/Prometheus®	Removal of circulating endotoxins and DAMPs, both albumin‐bound and water soluble	Phase 3 clinical trials	All aetiologies of liver disease and all precipitating events	No survival benefit in clinical trials	Refs. [39, 40]
	DIALIVE®	Removal of circulating endotoxins and DAMPs, both albumin‐bound and water soluble, combined with albumin infusion	Phase 2 clinical trial	Alcoholic cirrhosis and ACLF grades 1‐3a, any precipitating event of ACLF	More patients reached resolution of ACLF and with a faster time to resolution	NCT03065699
	Plasma exchange	Removal of circulating endotoxins and DAMPs	Phase 3 clinical trial	ACLF grades 1‐3a, All aetiologies of cirrhosis, all precipitating events	Results awaited	NCT03702920
Cellular Support	ELAD®	Allows impaired liver cells to recover	Phase 3 clinical trial	Severe alcoholic hepatitis	No improvement in survival	Ref. [41]
	MSCs	Promotes tissue repair	Phase 2 clinical trials	Predominantly viral‐related ACLF but also alcohol‐related ACLF	No improvement in survival but reduction in MELD scores	Ref. [42]
	HepaStem®	Immunomodulatory and antifibrotic effects	Phase 2 clinical trial	All causes of cirrhosis resulting in ACLF or acute decompensation	Safety demonstrated in phase 2 trial	NCT04229901
	Organoids	Mimic native liver function	Animal model	Drug‐induced liver failure model	Rescued animal livers from drug‐induced liver failure	Refs. [43, 44, 45, 46]

Abbreviations: ACLF, acute‐on‐chronic liver failure; ALP, alkaline phosphatase; AXL, anexelekto; DAMP, danger‐associated molecular pattern; ECLS, extracorporeal liver support; G‐CSF, granulocyte colony‐stimulating factor; HBV, hepatitis B virus; IL, interleukin; KCTD9, potassium channel tetramerisation domain containing 9; LPS, lipopolysaccharide; MELD, Model for End‐Stage Liver Disease; MERTK, Mer tyrosine kinase; MPO, myeloperoxidase; MSC, mesenchymal stem cell; NKG2D, natural killer cell group 2D; NSBB, non‐selective beta blocker; PAMP, pathogen‐associated molecular pattern; PD1, programmed cell death protein 1; PD‐L1, programmed death ligand 1; RIPK, receptor‐interacting protein kinase; ROS, reactive oxygen species; TIM3, T cell immunoglobulin and mucin domain‐containing protein 3; TLR, Toll‐like receptor; TRAIL, tumour necrosis factor‐related apoptosis‐inducing ligand.

## 2 LIVER DAMAGE IN ACLF

Hepatocyte cell death and liver damage play a major role in the development of ACLF. Damage‐associated molecular patterns (DAMPs) are molecules released from cells that are injured, dying or dead, such as high‐mobility group box 1 protein (HMGB1) and histones. DAMPs are increased in ACLF because of immunogenic forms of cell death that lead to release of cellular components, such as necrosis, necroptosis and pyroptosis, become predominant.^47^ Pathogen‐associated molecular patterns (PAMPs), highly conserved essential bacterial molecules, such as lipopolysaccharide (LPS), are also increased in ACLF due to infections and enhanced gut bacterial translocation. In the liver, DAMPs and PAMPs are recognised by various Pattern Recognition Receptors (PRRs) including Toll‐like receptors; for example, LPS is recognised by TLR4.^48^ TLR4 is expressed by multiple cell types, including monocytes, Kupffer cells and hepatocytes and upregulated hepatic TLR4 expression in ACLF may sensitise the liver to LPS‐driven tissue injury.^49^, ^50^, ^51^, ^52^ LPS binding to TLR4 induces pro‐inflammatory cytokine secretion, which triggers further inflammation, cell death and liver injury. The production of these pro‐inflammatory cytokines requires activation of the inflammasome, an intracellular multi‐protein complex that converts pro‐caspase‐1 to activated caspase‐1, which then converts pro‐interleukin‐1 beta (pro‐IL‐1*β*) to bioactive IL‐1*β*.^53^ IL‐1*β* contributes to the pro‐inflammatory response and chemokine production that results in the recruitment of neutrophils and monocytes to the liver and activation of Kupffer cells.^54^ IL‐1*α* is produced in response to a non‐canonical activation of the inflammasome. The importance of these cytokines in ACLF is demonstrated by the fact that single nucleotide polymorphisms in the IL‐1*β* and IL‐1 receptor antagonist (IL‐1ra) genes, resulting in reduced levels of circulating IL‐1*β* and IL‐1*α*, are protective against ACLF.^55^ Additionally, circulating levels of IL‐1*α* predict development of fatal ACLF in compensated cirrhotic patients, whilst circulating levels of IL‐1*β* predict ACLF development in recompensated patients.^56^

In the liver, neutrophil migration is responsible for hepatocyte cell death.^18^ Chemokine receptors, CXCR1 and CXCR2, expressed on neutrophils, are required for their chemotaxis to sites of liver injury.^57^ Higher expression of CXCR1 and CXCR2 has been found on neutrophils from patients with hepatitis B virus (HBV)‐ and alcohol‐related ACLF, which contributes to hepatocyte cell death and inflammatory cytokine production, including IL‐6 and IL‐8.^18^ Monocyte infiltration into the liver may perpetuate hepatic injury and secretion of pro‐inflammatory cytokines, while their in‐situ development to macrophages promotes resolution of tissue injury.^58^ In HBV‐related ACLF, increased intra‐hepatic natural killer (NK) cells contribute to hepatocyte cell death, partly through pathways involving various NK‐cell surface receptors.^20^, ^22^, ^59^

Finally, while regeneration is crucial for recovery of liver function after injury, it appears impaired in ACLF.^60^, ^61^ Hepatocyte proliferation capabilities are reduced, which is thought to be a result of decreased levels of growth factors produced by liver endothelial cells and liver mesenchymal stem cells.^61^

## 3 SYSTEMIC INFLAMMATION AND IMMUNE DYSFUNCTION IN ACLF

**FIGURE 1:**
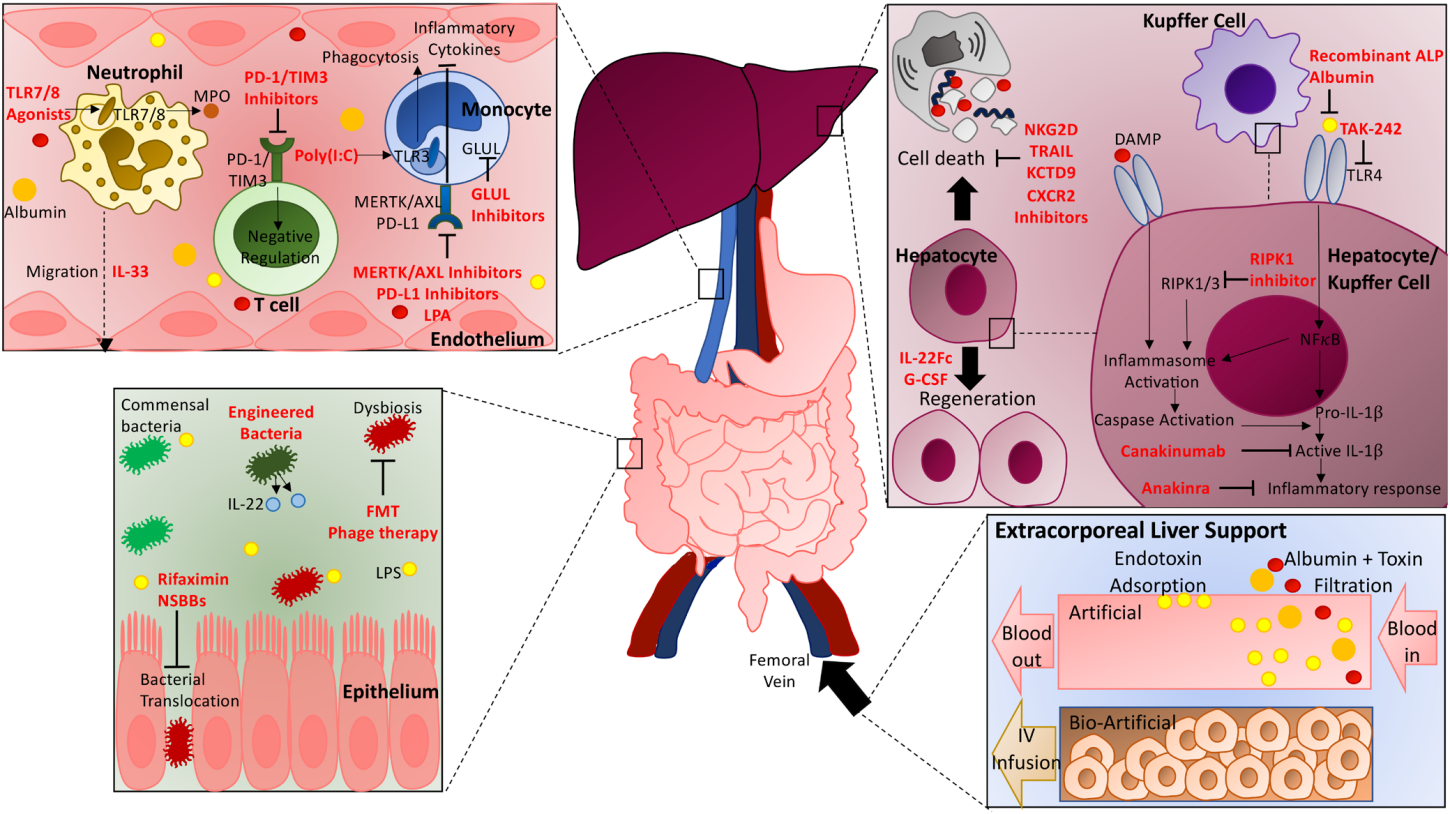
Mechanism of action of novel therapies for ACLF. Therapeutic approaches include targeting gut bacterial translocation and the microbiome, liver inflammation, cell death and regeneration, circulating immune cells and extracorporeal liver support. Dysbiosis in ACLF results in increased translocation of bacterial components and these PAMPs activate innate immune cells and thereby contribute to systemic inflammation. Antibiotics, NSBBs, engineered bacteria, FMT and phage therapy aim to improve dysbiosis and reduce bacterial translocation. Hepatocytes are injured by PAMPs and DAMPs and the resulting local inflammatory response. IL‐1 inhibitors and TLR4 inhibitors decrease this inflammation. RIPK1 inhibitors inhibit cell death and NKG2D, TRAIL, KCTD9 and CXCR2 inhibitors aim to reduce innate immune cell‐mediated hepatocyte death. IL‐22 and G‐CSF promote hepatic regeneration. Systemic inflammation and immune dysfunction contribute to organ failures and development of bacterial infections respectively. TLR7/8 agonists, IL‐33, PD1/TIM3 inhibitors, Poly(IC), MERTK/AXL inhibitors, LPA and glutamine synthetase inhibitors reduce circulating immunosuppressive responses. Extra‐corporeal liver support can be divided into artificial, such as Dialive or plasma exchange, and artificial, such as mesenchymal stem cells or organoids. Artificial support devices remove circulating toxins. Cellular therapies promote hepatic regeneration. ALP, alkaline phosphatase; AXL, anexelekto; DAMP, danger‐associated molecular pattern; FMT, faecal microbiota transplantation; G‐CSF, granulocyte colony‐stimulating factor; IL, interleukin; LPA, lysophosphatidic acid; LPS, lipopolysaccharide; MERTK, Mer tyrosine kinase; MPO, myeloperoxidase; NSBBs, non‐selective beta blockers; NKG2D, natural killer cell group 2D; PAMP, pathogen‐associated molecular pattern; PD1, programmed cell death protein 1; PD‐L1, programmed death ligand 1; TIM3, T cell immunoglobulin and mucin domain‐containing protein 3; TLR, Toll‐like receptor; TRAIL, tumour necrosis factor‐related apoptosis‐inducing ligand.

Systemic inflammation is suggested to be a key driver in the development of ACLF^51^, ^62^, ^63^, ^64^, ^65^ (Figure 1). Systemic inflammation is present in patients with cirrhosis and progresses in severity as cirrhosis advances from compensated to decompensated states to ACLF.^63^, ^64^ This intense systemic inflammation in ACLF is characterised by a large increase in plasma levels of pro‐inflammatory cytokines (IL‐6 and tumour necrosis factor (TNF)‐*α*), chemokines, adhesion molecules, soluble markers of macrophage activation (soluble cluster of differentiation 163 (sCD163) and mannose receptor), C‐reactive protein and circulating white blood cells.^1^, ^64^, ^66^, ^67^ Notably, the severity of the systemic inflammation in ACLF correlates with the degree of organ failure as well as survival.^64^, ^66^ Immunometabolism also plays an important role in the development of ACLF. Metabolomics analysis has shown that the intense systemic inflammation in ACLF is associated with major changes in metabolic pathways, including enhanced glycolysis and decreased mitochondrial beta‐oxidation.^68^ The resulting decrease in fatty acid‐based energy supply, combined with the mitochondrial reactive oxygen species (ROS) induced by inflammation, contribute to mitochondrial dysfunction and this may lead to metabolic suppression in peripheral organs, and therefore contribute to development of organ failures.^68^, ^69^

Alongside this intense systemic inflammation, there co‐exists an excessive anti‐inflammatory response in ACLF, characterised by functional defects of circulating immune cells and an increase in anti‐inflammatory mediators such as IL‐10, IL‐1ra and prostaglandin E2 (PGE2).^51^, ^63^, ^65^, ^66^, ^70^ This immunodeficiency makes patients prone to developing bacterial infections, which are highly frequent in ACLF and are associated with poor clinical course and high mortality.^71^

At a cellular level, there is an increased neutrophil count, and an elevated neutrophil‐to‐lymphocyte ratio predicts mortality in ACLF.^18^, ^72^ In decompensated alcohol‐related cirrhosis, a frequent trigger of ACLF, neutrophils show deficiencies in their functions of phagocytosis, myeloperoxidase (MPO) exocytosis and respiratory burst.^73^, ^74^ ROS production, which is required for the respiratory burst, is increased at a basal level in neutrophils of ACLF patients but is reduced in response to bacterial stimulation, highlighting an exhausted phenotype with impaired functions in bacterial defence.^27^, ^75^ Monocytes show reduced expression of HLA‐DR, leading to impaired antigen presentation.^32^ There is an expansion of immunosuppressive monocytic populations, including monocytes expressing MER receptor tyrosine kinase (MERTK+) and programmed death ligand 1 (PD‐L1+), as well as monocytic myeloid‐derived suppressor cells (M‐MDSCs).^28^, ^32^, ^76^ The latter suppresses bacterial uptake, TLR‐elicited inflammatory responses to microbial challenge and impair T cell function.^32^ T cells have increased expression of negative regulatory receptors including programmed cell death protein‐1 (PD‐1) and T cell immunoglobulin and mucin domain‐containing protein 3 (TIM3).^34^

This systemic inflammation may be triggered by PAMPs resulting from bacterial infections but can also derive from intestinal translocation of bacterial components. Worsening of liver disease is associated with intestinal bacterial overgrowth and dysbiosis, with reduced faecal microbial gene richness and species diversity.^77^, ^78^ Dysbiosis causes intestinal inflammation, which leads to increased pathological gut bacterial translocation and therefore contributes to systemic inflammation.^79^ Additionally, microbial metabolites correlate with the development of ACLF, and distinct microbiome profiles have been found in the stool and blood of patients with ACLF.^78^, ^80^, ^81^, ^82^

Interestingly, mechanisms involved in liver damage and systemic inflammation are different according to the triggering events of ACLF, implying that there may be different therapeutic targets based on the aetiology of ACLF. Bacterial infection‐induced ACLF leads to significant increases in TNF‐*α*, IL‐6 and IL‐1ra, whereas alcohol‐induced ACLF leads to a significant increase in IL‐8.^66^ In the same line, different triggers and underlying liver disease induce different patterns of immune response. For example, bacterial infections induce inflammation through recognition by dedicated PRRs as well as through virulence factors resulting in high levels of TNF‐*α*, IL‐6 and acute phase response proteins.^83^ In contrast, excessive alcohol consumption results in gut bacterial dysbiosis and increased intestinal permeability.^84^ In non‐alcoholic steatohepatitis, hepatic immune cells are activated by inflammatory mediators from adipose tissue and the gut.^85^ Infections trigger both innate and adaptive immunity, whereas DAMPs induce only innate inflammation.^86^ An ex vivo study found that neutrophil dysfunction in HBV‐related ACLF was influenced by the circulating environment and therefore one could speculate that the different circulating cytokine profiles in the various precipitating events of ACLF have different impacts on immune cells.^87^ Further evidence pointing to the heterogeneity of ACLF is that patients with alcohol‐related cirrhosis and active alcoholism who develop ACLF are younger, have more pronounced derangements in laboratory tests (leukocyte count, aspartate aminotransferase (AST) and bilirubin) and develop more severe grades of ACLF compared to patients who develop ACLF without active alcoholism.^1^, ^88^

## 4 THERAPIES TARGETING LIVER INFLAMMATION AND REGENERATION

### 4.1 Endotoxins – LPS/TLR4

TLR4 inhibitors have been proposed as a strategy to reduce hepatocyte injury.^52^ In a rodent model of ACLF, a small molecule TLR4 antagonist (termed TAK‐242)‐improved survival when given both pre‐ and post‐acute liver insult.^52^ This led to reduced liver and renal injury and circulating plasma levels of pro‐inflammatory cytokines. TAK‐242 had been planned for evaluation in phase 2 clinical trial in patients with acute alcoholic hepatitis resulting in ACLF, however, the trial was recently closed before recruitment started (NCT04620148). Of note, TAK‐242 has already been evaluated in a randomised controlled trial (RCT) in patients with severe sepsis, but failed to suppress cytokine levels and did not improve mortality.^89^ Alkaline phosphatase (ALP) is another potential therapeutic target acting on endotoxins. ALP is thought to detoxify LPS through dephosphorylation, therefore reducing its activity.^90^ Recombinant ALP has been studied in a rodent model of cirrhosis, where treatment prior to acute liver insult reduced hepatocyte death and hepatic cytokine expression.^15^ This suggests potential in preventing progression from decompensated cirrhosis to ACLF.

Human albumin is already in clinical use for prevention of circulatory dysfunction after large‐volume paracentesis or hepatorenal syndrome in spontaneous bacterial peritonitis.^5^ However, there are novel mechanisms being uncovered with regards to its beneficial immunomodulatory and anti‐inflammatory effects, and this could lead to an expansion of the clinical indications for use of albumin.^91^ Albumin has endotoxin‐binding capacity and endotoxin activity decreases in the presence of physiological albumin concentrations.^92^ Human albumin has also been proposed to reduce LPS‐induced signalling by internalising in leukocytes and inhibiting TLR signalling in endosomes.^93^ Alongside this, albumin reverts the PGE2‐induced immune dysfunction observed in patients with decompensated cirrhosis.^70^ In patient with decompensated cirrhosis, medium to long‐term (12 weeks to 18 months) albumin infusions result in reduced levels of circulating inflammatory cytokines, improve circulatory function and overall survival.^94^ However, short‐term (2 weeks) administration of albumin in a multi‐centre RCT in patients hospitalised in the context of decompensated cirrhosis did not improve the development of infection, kidney dysfunction or death.^16^, ^95^ The place of albumin infusion in the ACLF setting remains to be elucidated.

### 4.2 IL‐1

Targeting of IL‐1 cytokine could prevent the development of ACLF, and in a mouse model of alcohol‐related ACLF, treatment with anakinra, an IL‐1 receptor inhibitor, reduced liver inflammatory cell infiltration, hastened the downtrend of alanine aminotransferase (ALT) and increased markers of liver cell proliferation.^96^ Anakinra, in combination with pentoxifylline and zinc sulphate, did not lead to a significant improvement in survival in a randomised study compared to steroids.^97^ Another larger phase 2 RCT of anakinra with zinc sulphate in patients with severe alcoholic hepatitis was stopped early after a planned interim analysis as anakinra resulted in a lower 90‐day survival (NCT04072822).^98^ Canakinumab, a monoclonal antibody selectively inhibiting IL‐1*β*, has been tested in alcoholic hepatitis in a phase 2 RCT, where it led to histological improvements on liver biopsy but this did not translate into improved survival or Model for End‐Stage Liver Disease (MELD) score (NCT03775109).

### 4.3 Cell death pathways

Necroptosis is a form of cell death that becomes more frequent in the ACLF setting.^47^ It shares upstream markers with apoptosis, but ultimately results in cell swelling, rupture and DAMPs release. Receptor‐interacting protein kinase (RIPK) 1 and 3 are enzymes that play key roles in necroptosis. Serum levels of RIPK3 are higher in ACLF compared to non‐ACLF patients and correlate with severity and mortality.^17^, ^99^ In a rodent model of ACLF, pre‐treatment (i.e. prior to the acute insult leading to ACLF) with a RIPK1 inhibitor (NEC‐1) reduced total liver and kidney cell death, AST values and circulating histones.^99^ These results were validated in a second mouse model of ACLF with a different inhibitor of RIPK1, which was given post‐acute insult to the liver and also resulted in reduced liver cell death.^99^

Pyroptosis, another immunogenic form of cell death, where the protein gasdermin‐d plays a key role, has been shown to be important in the development of alcoholic hepatitis.^100^ This suggests the future potential of gasdermin‐d inhibitors in targeting this process in ACLF precipitated by alcoholic hepatitis.

### 4.4 IL‐33‐ST2‐CXCR2 pathway

CXCR1/2 contributes to apoptosis and necrosis of hepatocytes in vitro as well as production of inflammatory cytokines, suggesting that neutrophils play a role in hepatocyte death in ACLF.^18^ Inhibiting CXCR1/2 with an antagonist (Scheme 527123)‐reduced hepatocyte cell death and neutrophil ROS in vitro.^18^ Conversely, neutrophil migration to sites of infections is an important first line of defence. Patients with severe alcoholic hepatitis demonstrate altered signalling in the IL‐33/ST2 pathway in neutrophils and this results in reduced expression of CXCR2 on circulating neutrophils.^19^ The altered signalling in IL‐33/ST2 was associated with short‐term mortality and development of infections. Treatment of neutrophils with IL‐33 partially restored the signalling defects, CXCR2 expression and migration capacity.^19^ This highlights IL‐33 as a novel therapeutic agent. However, the beneficial and detrimental effects of modulating CXCR2 and therefore neutrophil migration must be carefully balanced to aid fighting infection but prevent worsening of liver damage.

### 4.5 NK cell receptors (TRAIL, NKG2D, and KCTD9)

The therapeutic targets under investigation related to NK cells have all been studied in HBV‐related ACLF, potentially because of the important role played by NK cells in the innate immune response against viral infections. Tumour necrosis factor‐related apoptosis‐inducing ligand (TRAIL) is part of the TNF super family which induce cell death. Activated NK cells express high levels of TRAIL, which results in apoptosis of hepatocytes through signalling on its corresponding receptor TRAILR. TRAIL expression is higher in peripheral NK cells in patients with HBV‐related ACLF compared to patients chronically infected with HBV.^20^ A TRAIL‐blocking antibody reduced NK cell‐mediated apoptosis of hepatocytes in vitro.^20^ In parallel, natural killer cell group 2D (NKG2D) receptors are expressed on NK cell membranes and they activate NK cells.^21^ NKG2D expression on NK cells is increased in HBV‐related ACLF compared to chronic hepatitis.^101^ Inhibiting NKG2D in vitro with a monoclonal antibody reduces inflammatory cytokine production (interferon‐*γ* (IFN‐*γ*), TNF‐*α*, perforin and granzyme B), but detrimentally reduces inhibition of HBV replication, which may limit its potential as a therapy.^101^ Finally, potassium channel tetramerisation domain containing 9 (KCTD9) is a protein expressed by NK cells which play a role in the regulation of NK cell function.^23^ KCTD9 expression is increased in both peripheral and hepatic NK cells of patients with HBV‐related ACLF, compared to patients with chronic HBV.^22^ Increased expression of KCTD9 leads to an increase in NK cell cytotoxicity towards hepatocytes and INF‐*γ* production. Inhibiting KCTD9 with short hairpin RNA results in reduced cytotoxic function and IFN‐*γ* production.^22^ These results have been extended to a viral fulminant hepatitis mouse model, where delivery of plasmid short hairpin RNA against KCTD9 improves survival and liver function and decreases histological liver damage.^102^ Further work is therefore needed on its role in ACLF.

### 4.6 IL‐22

IL‐22 is a pro‐regenerative cytokine which promotes hepatocyte proliferation.^103^, ^104^ High levels of serum IL‐22, or low ratios of IL‐22 binding protein (IL‐22BP) to IL‐22 (IL‐22BP/IL‐22), are strongly associated with the progression to ACLF and mortality.^24^ This elevated IL‐22 may be a compensatory role in response to the liver injury induced by ACLF. In a mouse model of ACLF, treatment with IL‐22 therapy, provided in the form of IL‐22Fc (two IL‐22 molecules linked to an immunoglobulin constant region), promoted liver repair and reduced bacterial infection.^25^ F‐652, another form of IL‐22Fc, has been studied in phase 2 clinical trial in alcoholic hepatitis.^105^ Results showed that F‐652 is safe and is associated with improvement in Lille and MELD scores. F‐652 also reduced markers of inflammation and increased markers of hepatic regeneration. These results warrant a phase 3 RCT to further assess the efficacy of F‐562.

### 4.7 Granulocyte colony‐stimulating factor

G‐CSF mobilises bone marrow CD34+ haematopoietic stem cells and promotes hepatic regeneration.^26^, ^106^ However, a multi‐centre placebo‐controlled phase 2 RCT found that G‐CSF did not improve survival, liver function scores or the occurrence of infections in ACLF.^107^ As a result, this study was stopped after interim analysis and G‐CSF is not recommended as a standard treatment for ACLF. However, in combination with TAK‐242 it improved the severity of liver injury in animal models of ACLF.^108^ Treatment with G‐CSF alone resulted in enhanced liver cell death and mortality but treatment with TAK‐242 prevented the increased mortality associated with G‐CSF treatment. The combination of both treatments was superior to both single treatments in reducing liver cell death. The proposed explanation is that the regenerative effects of G‐CSF are seen in an environment with reduced inflammation.

## 5 THERAPIES TARGETING SYSTEMIC INFLAMMATION AND ITS CONSEQUENCES ON THE IMMUNE SYSTEM

### 5.1 TLR7/8 pathway

Neutrophils from patients with decompensated alcoholic cirrhosis show impaired activation of a signalling pathway involving p38‐mitogen‐activated protein kinase (MAPK) and this results in reduced MPO release.^73^ In ex vivo experiments, a TLR7/8 agonist (CL097), which activates p38‐MAPK, improved MPO release and bactericidal activity against E. coli in neutrophils from these patients.^73^ CL097 also increased superoxide production following stimulation with the bacterial formylated peptide N‐formylmethionine‐leucyl‐phenylalanine (fMLP) both in isolated neutrophils and in whole blood.^27^ These results on superoxide production were reproduced with a second TLR7/8 agonist, R848.^27^ This suggests the potential for TLR7/8 agonists to restore antimicrobial responses in immunosuppressed states.

### 5.2 MERTK and AXL pathways

MERTK and anexelekto (AXL) are both TAM receptors, a family of receptors that inhibit TLR signalling pathways.^29^ Expression of MERTK on monocytes is associated with hepatic and extra‐hepatic disease severity scores and negatively correlated with pro‐inflammatory cytokine production by monocytes in response to LPS.^28^ Culture of isolated ACLF monocytes with a MERTK inhibitor (UNC569) up‐regulated the activation marker HLA‐DR and increased production of inflammatory cytokines in response to LPS.^28^ Therefore, MERTK inhibitors may be beneficial in eliminating immunosuppressive responses. Similarly, there is expansion of a population of AXL‐expressing monocytes in cirrhosis.^33^ Expression is associated with disease severity and 1‐year mortality.^33^ AXL‐expressing monocytes displayed reduced pro‐inflammatory responses to LPS and inhibited T cell proliferation. BGB324, also known as bemcentinib, a highly selective small molecule inhibitor of AXL, was originally developed as a cancer therapeutic target and is currently being evaluated in phase 2 trials in various cancers (NCT03184571 and NCT02872259). BGB324 improved LPS‐induced cytokine production from monocytes from patients with cirrhosis ex vivo, suggesting its potential for further investigation.^33^

### 5.3 Immune checkpoint pathways: PD‐L1/PD‐1 and TIM3

Peripheral monocytes from patients with decompensated cirrhosis show increased expression of PD‐L1 compared to compensated cirrhosis and healthy controls.^109^ Furthermore, patients who develop infections have a significantly higher percentage of monocytes expressing PD‐L1 compared to those who do not develop infections.^109^ Similarly, circulating monocytes from patients with ACLF who also develop sepsis show increased expression of PD‐L1.^76^ In a mouse model of chronic liver disease with superimposed infection, anti‐PD‐L1 antibody treatment reversed the immunosuppressive macrophage profile and reduced bacterial dissemination.^109^

PD‐1 is also being investigated as a therapeutic target. Peripheral monocytes from patients with acute liver failure (ALF) show increased PD‐1 expression and these PD‐1+ monocytes demonstrate an immunosuppressive phenotype.^110^ PD‐1 antibody blockade in vitro restored innate immune responses in monocytes from patients with ALF.^110^ Anti‐PD‐1 therapy has yet to be tested in an ACLF group, but given that lymphocytes from patients with decompensated cirrhosis and acute alcoholic hepatitis show a higher expression of PD‐1 compared to compensated cirrhosis or healthy controls, PD‐1 inhibitors may indeed prove beneficial.^34^, ^109^ Additionally, when PD‐L1+ monocytes from patients with ACLF and sepsis were co‐cultured ex vivo with T cells from healthy controls with LPS stimulation, the T cells developed increased expression of PD‐1 and TIM3.^76^ TIM3 expression is also increased on peripheral T cells from patients with alcoholic hepatitis, and in these patients, antibody co‐blockade of PD‐1 and TIM‐3 increased the numbers of bacterially‐challenged peripheral blood mononuclear cells (PBMCs) producing IFN‐*γ*, whilst reducing IL‐10‐producing PBMCs.^34^ This co‐blockade however did not provoke the production of pro‐inflammatory cytokines (IL‐1*β*, IL‐6, IL‐8 and TNF*α*) involved in systemic inflammatory responses. Ex vivo treatment of whole blood with anti‐PD1 and anti‐TIM3 blocking antibodies enhanced neutrophil phagocytosis and oxidative burst.^34^

### 5.4 Autotaxin–lysophosphatidic acid (LPA) pathway

Autotaxin (ATX) is upregulated in ACLF and this increases production of the bioactive lipid LPA from lysophosphatidylcholine (LPC).^30^ In monocytes from patients with ACLF‐cultured ex vivo, LPA treatment reduced pro‐regulatory phenotypes.^30^ This effect was seen predominantly through decreased MERTK and CD163 expression. LPA treatment also increased TNF‐*α* production by monocytes. Therefore, LPA seems to play a key role in the monocyte reprogramming that underlies immune paresis in ACLF, and hence modulation of immune responses by targeting LPA receptors is an important potential therapeutic strategy. Of note, ATX is elevated in non‐ACLF chronic liver diseases such as primary biliary cholangitis and pregnancy and is associated with fibrosis progression, which suggests it may be a potential therapeutic target in other chronic liver diseases.^111^, ^112^

### 5.5 Glutamine synthetase and ammonia pathways

Glutamine metabolism has been studied as a way to target the different metabolic requirements that develop in systemic inflammation in ACLF. Glutamine anabolism through glutamine synthetase has been suggested to contribute to the sustained suppressive phenotype of ACLF monocytes.^31^ Monocytes from patients with alcohol‐related ACLF demonstrate an elevated glutamine synthetase (GLUL) to glutaminase (GLS) ratio, which correlates with MELD score.^31^ An inhibitor of GLUL, methionine sulfoximine (MSO), improved ACLF monocyte capacity to recognise/engulf bacteria, inhibited IL‐10 production and promoted production of TNF‐*α*.^31^ Of note, ammonia also plays a role in the metabolism of glutamate, in that GLUL combines ammonia and glutamate to form glutamine. Therefore, a concern with inhibiting GLUL is hyperammonaemia, and subsequent hepatic encephalopathy (HE). Ammonia causes disturbances in the central nervous system, for example cellular swelling and neuroinflammation and contributes to neuronal cell death and mitochondrial dysfunction.^113^, ^114^

## 6 THERAPIES TARGETING GUT‐BACTERIAL TRANSLOCATION

### 6.1 Antibiotics and statins

Rifaximin, a poorly absorbed antibiotic, is already used clinically to prevent recurrent hepatic encephalopathy. It may slow the progression of liver disease through regulating the gut microbiome, decreasing bacterial translocation, reducing circulating endotoxin and decreasing portal pressure.^115^, ^116^, ^117^ However, results are conflicting with other studies showing minimal effect on the gut microbiome and inflammatory markers.^118^, ^119^ A multicentre phase 2 RCT assessed the safety of combined therapy with rifaximin and simvastatin in preventing ACLF, which showed safety of low‐dose simvastatin (20 mg/day) (NCT03780673).^120^ This dose will be assessed in a phase 3 trial (NCT03780673). Statins have indeed anti‐inflammatory and anti‐fibrotic properties but also show microbiome‐diversifying effects and improve intrahepatic resistances and portal hypertension. Simvastatin treatment prevents ACLF development in animal models.^121^, ^122^, ^123^ A recent large retrospective cohort study found that statin use in patients with cirrhosis was associated with reduced risk of development of ACLF.^124^

### 6.2 Non‐selective beta‐blockers

Non‐selective beta‐blockers (NSBBs), which inhibit the actions of catecholamines through targeting both *β*1 and *β*2 adrenoreceptors, are already in clinical use for primary and secondary prophylaxis of variceal bleeding, where their clinical benefit comes from a decrease in portal pressure.^125^ However, NSBBs are also thought to have other beneficial effects, including reducing bacterial translocation and therefore alleviating systemic inflammation.^126^, ^127^ In keeping with this, noradrenaline levels are up to three times higher in ACLF than in patients with an acute decompensation of cirrhosis, and this is associated with the systemic inflammatory response and brain and kidney function in ACLF.^128^ Two observational studies found reduced 28‐day mortality in ACLF patients who received NSBBs compared to those who did not receive NSBBs.^35^, ^36^ However, the use of NSBBs in clinical practice may be limited by hypotension requiring inotropic support. Additionally, the evidence for NSBBs in ACLF comes from observational studies, which introduce much higher risk of bias compared to RCTs.

### 6.3 Probiotics and engineered bacteria

Probiotics are live bacteria that provide health benefits for the host, and RCTs in patients with cirrhosis have demonstrated improved liver function and reduced risk of HE but no effect on immune function.^129^, ^130^ Newer probiotics are now in development which consist of engineered bacteria with the selection of genes that result in beneficial effects, such as improved colonisation, immunomodulation or host‐bacteria interaction.^131^ For example, *Lactobacillus reuteri* engineered to produce IL‐22 prevented gut bacterial translocation and reduced alcohol‐induced liver injury and inflammation in a mouse model of alcohol‐induced liver disease.^37^

### 6.4 Faecal microbiota transplantation

FMT from healthy donors to patients is a strategy to restore the diversity of the patient's microbiome. It may also restore gut barrier function and thereby reduce bacterial translocation. In a pilot study, severe alcoholic hepatitis patients who received FMT had improved survival compared to historical controls who received standard of care.^132^ It is currently being investigated in an RCT comparing FMT to corticosteroid therapy in patients with severe alcoholic hepatitis (NCT03091010). However, one concern with FMT is the risk of infections with some studies reporting an increased risk of SBP and transmission of multi‐drug resistant bacteria.^133^

### 6.5 Phage therapy

Phages are viruses that infect bacteria and phage therapy has been proposed as a way of targeting specific bacterial enzymes in the gut microbiome.^134^ For example, the toxin cytolysin, that is secreted by *Enterococcus faecalis*, correlates with mortality and liver disease severity in patients with alcoholic hepatitis.^38^ Oral treatment with phages targeting cytolysin‐positive *E. faecalis* reduced alcohol‐induced liver injury and inflammation in humanised mice that were colonised with bacteria from the faeces of patients with alcoholic hepatitis.^38^ More work is needed to translate these results into humans, but it is hoped that ultimately phage therapy could lead to a precision medicine approach where an analysis of the patient's microbiome and metabolites could indicate which specific bacterial metabolic pathways could be targeted by phages.^133^

## 7 EXTRA‐CORPOREAL LIVER SUPPORT

### 7.1 Artificial support devices

#### 7.1.1 MARS ^®^ and Prometheus^®^

Targeting circulating inflammatory factors such as endotoxin and products of cell death is the basis of artificial extra‐corporeal liver support therapies. Molecular Adsorbent Recirculating System (MARS)^®^ and Prometheus^®^ are both liver support devices which are based on an albumin‐dialysis system that selectively eliminates both albumin‐bound and water‐soluble toxins. The two major RCTs that have assessed these devices in ACLF found no survival benefit compared to standard medical therapy.^39^, ^40^ A meta‐analysis comparing intensity of MARS treatment found that patients who received more than 4 MARS sessions (the high‐intensity treatment group) had improved survival compared to patients who received less than 4 or who did not receive MARS sessions (the low‐intensity treatment group).^135^ However, these results must be taken with caution as the ability to undergo MARS sessions could be closely related to the course of ACLF, therefore selecting the improvers in the high‐intensity group.

#### 7.1.2 Dialive™

Another type of extra‐corporeal liver support device called Dialive™ is currently under investigation. Dialive™ is a system that can be delivered using a kidney dialysis machine and uses two filters – one removes circulating products of liver failure, such as products of cell death and endotoxin, and the other removes dysfunctional albumin. This is combined with infusion of fresh albumin. In a pig model of ALF, it resulted in a reduced risk of death compared to controls.^136^ It has been investigated in a phase 2 RCT where patients with alcoholic cirrhosis and ACLF grades 1‐3a were randomised to receive either 3 to 5 Dialive sessions or standard care. Preliminary results have demonstrated that significantly more patients in the Dialive group reached resolution of ACLF and with a faster time to resolution, supporting further evaluation in phase 3 trials (NCT03065699).

#### 7.1.3 Plasma exchange

Plasma exchange (PE) is a well‐established technique that involves extracorporeal separation and removal of a patient's plasma and return with replacement plasma fluid. Its proposed benefit in ACLF is through removing circulating cytokines, albumin‐bound and water‐soluble toxins, such as endotoxin, bilirubin and ammonia and thereby reducing systemic inflammation.^137^ In a retrospective study of patients with HBV‐associated ACLF, it was suggested that receiving a minimum of 3 PE sessions improves both 90‐day and 5‐year survival compared to standard care.^138^ Another study in a group of patients with predominantly HBV‐associated ACLF observed that PE combined with plasma bilirubin adsorption improves survival compared to PE alone.^139^ PE with human serum albumin 5% is currently being investigated in the APACHE trial, a multi‐centre phase 3 trial investigating PE in patients with ACLF grades 1‐3a. Patients are randomised to receive PE, with the number of sessions dependent on the pattern of response to the PE, or standard medical therapy. This trial should determine whether PE is of benefit to patients with ACLF (NCT03702920). Until these trials are reported no extracorporeal devices can be recommended fully in ACLF.

### 7.2 Bio‐artificial and cellular therapies

#### 7.2.1 ELAD^®^

Cell‐based therapies have been proposed as a model to support liver function in ACLF. Extracorporeal cellular therapy (ELAD^®^) with hepatoblastoma‐derived C3A cells has been the only cell‐based therapy to date to have been studied in a phase 3 RCT, which assessed its efficacy in patients with severe alcoholic hepatitis.^41^ However, the trial failed to show an improvement in survival.

#### 7.2.2 Mesenchymal stem cells

Mesenchymal stem cells (MSCs) are cells that can self‐renew and differentiate into multiple different cell types, including hepatocytes.^140^ MSCs are thought to create a regenerative environment and promote tissue repair in damaged tissues.^141^ A recent meta‐analysis of 12 RCTs evaluated MSC therapy for patients with ACLF.^42^ The majority of patients in the analysis had viral‐related ACLF but there were also some patients with alcohol‐related disease. The meta‐analysis found that MSC therapy did not improve survival but was associated with a reduction in MELD score at 4, 12 and 24 weeks and improved albumin level at 4 and 24 weeks.^42^ Bone marrow‐ and umbilical cord‐derived MSCs showed similar efficacy in improving liver function.^42^

#### 7.2.3 Human allogeneic liver‐derived progenitor cells

Another potential cellular therapy for ACLF is human allogeneic liver‐derived progenitor cell (HALPC) therapy. HALPCs are obtained from the parenchyma of healthy human liver tissue and have immunomodulatory and anti‐fibrotic functions.^142^, ^143^ HepaStem^®^, a suspension of HALPCs, has been tested in an open‐label Phase 2 Trial in patients with ACLF (NCT04229901). Results showed safety of HepaStem treatment and supported further study to evaluate safety and efficacy.^144^

#### 7.2.4 Organoids

Rapid advances in liver bioengineering and regenerative medicine hold potential for future therapies in ACLF. Organoids are self‐organised 3D structures, grown in culture from stem cells or primary tissue, that mimic native tissue architecture and function.^145^ Multiple types of liver organoids have been successfully transplanted and rescued animal livers from drug‐induced failure.^43^, ^44^, ^45^, ^46^ However, further work is needed regarding the vascularisation of the organoids as well as the tumorigenic potential, in long‐term animal studies before translating to humans.^146^ Work will also be required to expand these organoids to human size. In the future, it is hoped that these organoids could generate patient‐derived bioengineered grafts that could be returned to the patient.^146^

## 8 FUTURE DIRECTIONS

Many of the therapies that have reached human trials have failed despite positive preclinical results. There may be two major reasons for this. Firstly, animal models of ACLF do not capture the diversity and complexity of the heterogenous ACLF syndrome in humans. Animal models have been developed by inducing both chronic and acute liver injuries. Chronic injury is most often generated by the injection of carbon tetrachloride (CCl_4_) or by bile duct ligation surgery, while acute injury is mostly generated by the injection of LPS, often in combination with D‐galactosamine hydrochloride (D‐GaIN). However, the major weakness of these models is that ACLF is significantly more complex in humans, with multiple precipitating events, complications and organ failures. For this reason, there is a need to improve these models by increasing their complexity. One example of this is the model developed by Xiang et al. which combines three stages: chronic liver injury with CCl_4_ injection, acute liver injury with a double dose of CCl_4_, and systemic bacterial infection with injection of *Klebsiella pneumoniae*.^25^ This model has closer features to ACLF than a two‐stage model, with higher mortality rates, serum bilirubin and extrahepatic organ injury. Future testing of new therapies may need to involve different models, which could help to identify roles in different disease courses of ACLF, although would significantly extend the length of the research process. These barriers to research with animal models may mean that future research will need to focus on ex vivo approaches that lead to early human experimental medicine studies.

Secondly, single‐agent therapy may not be sufficient to target the complex pathogenesis of ACLF. Evidence for this comes from the benefit of combination therapy with TAK‐242 and G‐CSF.^108^ This combination therapy targeted multiple aspects of the pathogenesis, including inflammation, cell death and inadequate regeneration. Furthermore, the findings suggested that the pair were not only additive but synergistic in their effects. A combination approach could also mean that unwanted side effects of one drug could be targeted by the other, for example, G‐CSF may prevent impaired response to pathogens induced by TLR4 inhibition.^108^ This theory may also explain the recent failure of anakinra in alcoholic hepatitis, given that this drug is known to target only the IL‐1 pathway. Therefore, future research initiatives may need to focus on combination therapy approaches targeting pathways from different aspects of the pathophysiology, for example, a combination targeting both inflammation and regeneration. This review outlines numerous preliminary targets that could be involved in a multi‐target approach.

## 9 CONCLUSION

This review provides a comprehensive overview on novel perspectives for the treatment of ACLF. The majority of these potential treatments are still in preclinical or early clinical stages of development. The identification of the roles of various immune cell subsets and inflammatory mediators in the pathogenesis of ACLF has provided us with a clearer understanding of disease development and has therefore allowed for the investigation of new therapeutic targets.

One challenge to developing therapies is that on one hand, there is systemic inflammation that drives organ failures, but on the other hand, bacterial infections are the most common precipitant of ACLF and a highly frequent cause of death, which is a result of immune exhaustion. Therefore, any therapy should modulate the immune system and target specific components of the immune response. Another challenge is the heterogeneity within the ACLF syndrome. Differences in the aetiologies of chronic liver disease, precipitating events and mechanisms of organ failures create a diverse ACLF patient group with variations in underlying pathophysiology. This heterogeneity will need to be considered when studying novel therapeutic agents.

Further human studies are needed to assess these novel therapies and determine their safety and efficacy. In the future, the combination of drugs targeting different aspects of the pathophysiology, and advanced organ support techniques may ultimately lead to better outcomes in ACLF patients.

## FUNDING INFORMATION

NIHR supported MM salary as an academic foundation trainee. FA was supported by an EASL Joan Rodes Fellowship.

## CONFLICT OF INTEREST STATEMENT

The authors declare no conflicts of interest related to this work.
